# Surveying the Knowledge and Practices of Health Professionals in China, India, Iran, and Mexico on Treating Tuberculosis

**DOI:** 10.4269/ajtmh.15-0538

**Published:** 2016-05-04

**Authors:** Steven J. Hoffman, G. Emmanuel Guindon, John N. Lavis, Harkanwal Randhawa, Francisco Becerra-Posada, Masoumeh Dejman, Katayoun Falahat, Hossein Malek-Afzali, Parasurama Ramachandran, Guang Shi, C. A. K. Yesudian, for the Research to Policy and Practice Study Team

**Affiliations:** Global Strategy Lab, Faculty of Law, University of Ottawa, Ottawa, Ontario, Canada; Centre for Health Economics and Policy Analysis, Department of Clinical Epidemiology and Biostatistics, McMaster University, Hamilton, Ontario, Canada; McMaster Health Forum, McMaster University, Hamilton, Ontario, Canada; Pan American Health Organization, Washington, DC; Department of Mental Health, Johns Hopkins Bloomberg School of Public Health, Baltimore, Maryland; Deputy of Research and Technology, Ministry of Health and Medical Education, Tehran, Iran; Department of Epidemiology and Biostatistics, School of Public Health, Tehran University of Medical Sciences, Tehran, Iran; Valmar International, Mumbai, India; Department of Policy Research, Chinese Peasants' and Workers' Democratic Party, Beijing, China; Health Systems Consultant and Trainer, Mumbai, India

## Abstract

Research evidence continues to reveal findings important for health professionals' clinical practices, yet it is not consistently disseminated to those who can use it. The resulting deficits in knowledge and service provision may be especially pronounced in low- and middle-income countries that have greater resource constraints. Tuberculosis treatment is an important area for assessing professionals' knowledge and practices because of the effectiveness of existing treatments and recognized gaps in professionals' knowledge about treatment. This study surveyed 384 health professionals in China, India, Iran, and Mexico on their knowledge and practices related to tuberculosis treatment. Few respondents correctly answered all five knowledge questions (12%) or self-reported performing all five recommended clinical practices “often or very often” (3%). Factors associated with higher knowledge scores included clinical specialization and working with researchers. Factors associated with better practices included training in the care of tuberculosis patients, being based in a hospital, trusting systematic reviews of randomized controlled double-blind trials, and reading summaries of articles, reports, and reviews. This study highlights several strategies that may prove effective in improving health professionals' knowledge and practices related to tuberculosis treatment. Facilitating interactions with researchers and training in acquiring systematic reviews may be especially helpful.

## Background

Educators, researchers, practitioners, and policymakers are increasingly cognizant and concerned that findings from research evidence are often not being put into action.^1^,^2^ A growing body of literature continues to demonstrate that research evidence is not consistently disseminated to health professionals who require it to inform their clinical practice and improve their patients' well-being. This knowledge deficit may result in substandard care, ineffective service provision, inefficient resource use, and further inequities in health outcomes. This reality is especially overwhelming for low- and middle-income countries (LMICs), which suffer greater limitations in resources compared with high-income countries. This situation is especially concerning when cost-effective interventions exist to address global health challenges but are simply not being fully or appropriately used.^3^

This gap between research evidence and clinical practices is particularly salient for improving tuberculosis control because of the proven effectiveness of treatments (i.e., combinations of first-line tuberculosis drugs)^4^ and past findings of suboptimal knowledge on this topic.^5^–^32^ Despite recent progress in controlling tuberculosis and achievement of the Millennium Development Goal (MDG) target to reverse the epidemic by 2015,^4^,^33^ there remains a pressing need for improvement given documented observations such as the disparities in regional progress^33^; the potential for 10-fold returns in economic growth on investment^34^; the disproportionate burden carried by those least able to manage it^33^; and the health gains that can be achieved by continued progress.^33^ The lethal, airborne nature of tuberculosis also poses a serious threat to global health security, particularly as some forms of the disease are already “virtually untreatable” (i.e., multidrug-resistant tuberculosis)^33^ and become increasingly so with inappropriate medicines use.^35^ Indeed, one of the greatest contributors to drug-resistant forms of tuberculosis is improper treatment that may be prescribed or administered by health professionals,^35^ either because of inaccurate diagnosis, insufficient treatment supplies, or limits in their knowledge of evidence-based tuberculosis control practices.^28^ In this way, the knowledge and practices of health professionals related to tuberculosis affect not only their individual patients, but also the global population as a whole. This situation requires a better understanding and an urgent response, lest the problem be allowed to persist or escalate.

This study aims to examine the gap between what is known internationally through research evidence about tuberculosis treatment interventions and the related knowledge and practices of health professionals in LMICs. This analysis is unique in that it is broad, uses methodology informed by a rich pool of evidence, and goes beyond descriptive analysis (e.g., makes use of logistic regression). It aims to identify determinants of health professionals' tuberculosis treatment knowledge and practices across sectors and in multiple countries. Recent studies from LMICs have been characterized by a focus on descriptive indicators^5^–^28^ and/or a limited context due to focused analysis of a single country or sector.^5^–^25^,^29^–^32^ In contrast, the wide-lens approach used in this study facilitates the identification of factors that may be more broadly generalizable and that can be targeted in larger global and regional efforts that are undertaken simultaneously in many countries.

## Methods

This study was part of a larger research effort sponsored by the World Health Organization, which aimed to assess the link between research, practice, and policy. Other components of this undertaking include efforts to examine whether and how health professionals make use of research evidence,^36^,^37^ the extent to which researchers support its use,^38^,^39^ and an assessment of health professionals' knowledge and practices related to malaria prevention and the use of insecticide-treated nets.^40^ This component of the investigation follows a similar methodology as the latter, and is the first analysis and presentation of the collected data on health professionals' knowledge and practices related to tuberculosis.

### Questionnaire design.

A questionnaire based on nine existing instruments was administered to respondents to identify items such as individual characteristics, working context, training, networking activities, access to, trust in, and use of research evidence.^41^–^49^ The survey also included five multiple-choice questions that tested participating health professionals' knowledge of tuberculosis treatment, and five questions used to assess relevant clinical practices.^50^–^55^ The instrument demonstrated high internal consistency (i.e., reliability) and content and face validity. It was administered in English in India and translated into Mandarin, Persian, and Spanish for administration in China, Iran, and Mexico, respectively. The development, translation, pilot-testing, reliability, and validity of this instrument are described elsewhere.^36^

### Data collection.

Country teams were situated in China, India, Iran, and Mexico. These LMICs differ with regard to population size, per capita income, health expenditures, life expectancy, tuberculosis prevalence, and computer and Internet access (Table 1).^56^–^59^ Each team administered the questionnaire locally between October 2004 and December 2005 with the aim of collecting complete responses from 100 health professionals in each country. Systematic reviews (with the Cochrane Library identified as the most comprehensive source of them) are emphasized in the current analysis. Widely acknowledged as the optimal approach to synthesizing global research evidence, systematic reviews offer summary information to support decision-making in an efficient way and are extensively available and internationally authoritative on clinical interventions,^60^–^62^ including directly observed therapy for the treatment of tuberculosis.^63^,^64^ The decisions to differentiate between scientific journals from high-income countries and the respondents' own countries and between full reports and summaries were suggested by previous studies.^37^,^43^,^65^

In China, the country team constructed a sampling frame using an existing list of tuberculosis control centers in the Hebei province surrounding Beijing. Weichang county and then 97 facilities within it were randomly selected after being stratified by the administrative authority (i.e., county, township, and village). Using a cluster random sampling process, 120 health professionals who provided care to patients with tuberculosis were sampled.

In India, a sampling frame was constructed from a preexisting list of health facilities set up by the Greater Mumbai Health Department. Health posts were stratified by density (i.e., ≤ 6, 7–9, or ≥ 10 health posts within a municipal location area) and then randomly selected. The chief medical officers of 100 health posts in the poor areas of Mumbai were sampled using a stratified cluster random sampling process.

In Iran, the country team constructed a sampling frame using a preexisting human resources database from each province's medical sciences university. Eleven health districts were randomly selected for sampling. Within these, 128 facilities were randomly chosen for sampling and stratified by geographic location (i.e., Sistan and Baluchetan province, Golestan province, and Hormozgan province) and type of profession (i.e., government general practitioner [GP], private GP, and specialist). Using a stratified cluster random sampling process, 128 primary care physicians and specialists providing care to tuberculosis patients were sampled.

Finally, in Mexico, the country team constructed a sampling frame using five distinct sources (i.e., physicians working for the Instituto Nacional de Enfermedades Respiratorias, physicians working for the Instituto de Seguridad y Servicios Sociales de los Trabajadores del Estado, physicians working for the Secretaria de Salud [Ministry of Health] in five facilities of Mexico City, physicians attending a training course at the Instituto Mexicano del Seguro Social, and private physicians involved in tuberculosis care attending a training course). A simple random sampling process was used to select 123 health professionals providing care to tuberculosis patients in Mexico City and the states of México, Nuevo León, and Jalisco.

### Data analysis.

In addition to the calculation of basic descriptive statistics for relevant items, simple ordinal logistic models were conducted in the exploration of factors associated with health professionals' knowledge and/or practices relevant to tuberculosis treatment.

Composite knowledge and practice scores were constructed for each respondent. Knowledge scores were composed of the proportion of the five multiple-choice knowledge-testing questions answered correctly, with each question weighted equally and no penalty awarded for incorrect answers.^54^,^55^ Composite practice scores were composed in a similar manner and were based on each professional's reported frequency of engaging in the five practices listed on a 5-point scale (i.e., 1 = “never,” 2 = “rarely,” 3 = “sometimes,” 4 = “often,” and 5 = “very often”). As one of the practices defined is contrary to recommended practice, the scale was inverted for one of the five items. Each question held equal weighting, resulting in individual practice scores calculated as integers ranging from 5 to 25.

The knowledge and practice scores were converted into quintiles within each country to be used as ordinal variables. The independent variables in both ordinal logistic models included health professionals' 1) use of particular sources of evidence, 2) views and activities related to improving clinical practice, and 3) individual and practice characteristics. Multiple imputations were used to fill in missing values using multivariate normal regressions with the use of 100 imputations. The implementation of this method was based on its ability to accommodate arbitrary missing value patterns.^66^ In cases where the dependent variable was missing, the observation was excluded from the model. All statistical analyses were conducted using Stata/MP 11.2 for Mac.

## Results

In total, 471 health professionals were approached for this study and 384 participated, yielding an overall response rate of 82%. Individual country response rates were 97% in China (*N* = 116/120), 100% in India (*N* = 100/100), 84% in Iran (*N* = 108/128), and 49% in Mexico (*N* = 60/123).

The majority of participating health professionals were male (65%), trained as general practitioners (81%), able to read and write English (52%), and worked in government-operated facilities (95%) and community health centers (55%). The respondents, on average, were 40 years of age and allocated the majority of their time to clinical practice (63%), as opposed to research (5%), teaching (10%), and administrative tasks (17%). Few respondents had easy access to a personal computer with a CD-ROM (22%) or the Internet (20%). Very few had earned masters or doctorate degrees (4%), and only a quarter (24%) worked with researchers in improving their clinical practice or quality of working life. Although many respondents had received training specifically in the care of tuberculosis patients since their last degree (79%), significantly less self-reported training on acquiring (3%) or appraising (6%) research evidence in the form of systematic reviews. Similarly, very few respondents self-reported using the electronic Cochrane library over the past 12 months (6%). Many more respondents did, however, self-report reading electronic or paper versions of clinical practice guidelines, protocols, or decision-support tools (55%), scientific journals either from their own country (58%) or high-income countries (18%), and summaries of articles, reports, and reviews from public and not-for-profit health organizations (56%) over the 12 months before data collection. Most respondents also indicated that research performed in their own country was of above average or excellent quality (56%), but that a higher quality of available research is important or very important to improve their work (78%) (Table 2).

Only very few respondents correctly answered all five knowledge questions regarding tuberculosis care (12%), whether from China (5%), India (5%), Iran (19%), or Mexico (22%). The range of correct responses for any individual question among all countries extended from 13% on the major side effects of isoniazid therapy (question 3) to 98% on the identification of first-line therapies for tuberculosis (question 2). The greatest variation for correct response rates within a single country was in India (13–98%), whereas the least variation was seen in Iran (55–93%). Most health professionals correctly identified that the minimum duration of therapy for culture-proven active tuberculosis is 6 months (87%), knew that a patient with three negative acid-fast bacilli sputum smears in three consecutive days is the best indicator for a noninfectious patient (83%) and were able to identify a tuberculosis therapy that was not a first-line treatment amid four first-line treatments (87%). However, less than half of all respondents correctly identified factors accelerating progression from tuberculosis infection to disease (44%) and the major side effect of isoniazid therapy (45%) (Table 3).^54^,^55^

Few professionals self-reported performing “often” or “very often” all five of the recommended clinical practices over the 12 months before data collection (3%). This trend was apparent whether respondents were from China (0%), India (7%), Iran (2%), or Mexico (5%). Many respondents reported often or very often: ensuring that treatment of new active tuberculosis patients was taken in the presence of a health worker for at least 2 months (81%) (while directly observed therapy is considered a recommended practice, it has not been proven to demonstrate a significant treatment effect in patients with tuberculosis or HIV^64^,^67^); providing education on the importance of taking medication regularly before initiating treatment of new active tuberculosis patients (88%); and notifying health authorities about new active tuberculosis patients (85%). It is also good that only few reported that they often or very often prescribed a treatment regimen of 5 months or less when treating new active tuberculosis patients (5%), which is contrary to recommended practice. However, only a few respondents self-reported often or very often recommending preventive chemotherapy for tuberculosis when treating HIV-infected patients (19%), which is a recommended practice. It is possible that this low proportion could be a result of a lack of knowledge or resources in less-resourced settings (Table 4).

The first ordinal logistic model identified two statistically significant factors associated with health professionals' knowledge scores related to tuberculosis treatment: 1) being a specialist physician (odds ratio [OR] = 2.84, 95% confidence interval [CI] = 1.15–7.05) and 2) working with researchers to improve their clinical practice or quality of working life (OR = 1.48, 95% CI = 1.01–2.17). The second ordinal logistic model revealed several factors that were associated with health professionals' practices related to tuberculosis treatment: 1) training relating to the care of patients with tuberculosis since their last degree (OR = 2.63, 95% CI = 1.05–6.60); 2) being based in a hospital (OR = 1.35, 95% CI = 1.18–1.54); 3) trusting somewhat or completely a systematic review of randomized controlled double-blind trials (OR = 1.78, 95% CI = 1.36–2.35); and 4) reading electronic or paper versions of summaries of articles, reports, and reviews from public and not-for-profit health organizations (OR = 1.42, 95% CI = 1.05–1.92) (Table 5).

## Discussion

### Principal findings.

There is significant room for improving knowledge and practices related to tuberculosis among the health professionals surveyed in this study. Only 45 of 384 health professionals (11.7%) correctly answered all five knowledge-testing questions, and only 12 of 384 professionals (3.1%) often or very often followed all five recommended practices. These disappointing results are not dissimilar to those previously documented in the literature.^5^–^32^,^68^ Indeed, findings of substandard knowledge and practices related to tuberculosis may just be examples of the broader gaps that exist in health professionals' knowledge and practices across diseases and conditions in low-, middle-, and high-income countries.^69^–^113^ While there may be many associated factors contributing to the knowledge and practices of these professionals, these widespread findings of suboptimal knowledge may also point to gaps in foundational and/or continuing education or training (e.g., reviewing local guidelines) in health professionals across different regions. In addition, a contributing factor for poor practice scores among the population we surveyed may be a shortage or maldistribution of medical personnel, as is observed in many regions worldwide.^114^,^115^

This study revealed several factors that may be associated with health professionals' knowledge and/or practices in the treatment of tuberculosis. For example, respondents who received specialist training demonstrated better knowledge but not better practices than those who did not. The association between clinical specialization and level of knowledge and practice behaviors has been previously explored, but with varying results across different specialties and a paucity of consolidated findings, pointing to an area for future research.^35^,^116^,^117^ Similarly, interactions with researchers to improve clinical practice or quality of working life were found to be associated with higher knowledge among participating health professionals, but not better practices. Interestingly, this same factor was found to be associated with better practices and not knowledge among a sample of professionals surveyed on their practices in malaria prevention,^40^ which supports findings in previous research literature.^118^,^119^ It is important to note that there may be a variety of perceived barriers hindering health professionals from becoming more involved in research, and mitigating these impedances could be an effective means to improve interactions between clinicians and researchers. These include, but are not limited to, inadequate training in research methods, paucity of relevant opportunities and collaborators, community distrust of research, and lack of time.^120^,^121^

Although it is possible that the factors under study may affect knowledge and practices by different means, the divergent results found in this study may also be partially explained by social desirability bias. Presumably, social desirability bias would affect the self-reporting of practices a great deal more than it would affect the answers to knowledge-testing questions. Other possibilities explaining these results could be that the models may have lacked sufficient power, the dependent variables may not be satisfactory reflections of professionals' real knowledge and/or practices, and there may be confounding variables skewing the current analyses.

### Strengths and limitations of the study.

There are five principal strengths of this study. First, the study investigated knowledge and practices related to tuberculosis, a disease that is prioritized by the MDGs^4^,^33^ and has proven, effective treatments.^4^ Second, the survey instrument was constructed from existing questionnaires and was pilot tested and assessed for both reliability and validity, ensuring a rigorous methodological process.^36^ Third, data were collected from four different LMICs that are distinguishable in population, life expectancy, prevalence of tuberculosis, and other characteristics. Fourth, high response rates were achieved in three of the four countries surveyed (i.e., China, India, and Iran). Finally, the knowledge and practice composite scores used in the analysis were calculated from a variety of testing questions for which respondents were not given the correct answer.^54^,^55^ This measure maintained more objectivity than using a self-evaluation where health professionals would be asked to assess whether they had “high” or “low” levels of knowledge and practices, which is a metric seen in past studies.

This study also has at least five limitations. First, despite professional translation of the English-language questionnaire into Mandarin, Persian, and Spanish for administration in China, Iran, and Mexico, respectively, linguistic and cultural differences between countries may have affected respondents' interpretations of particular questions. Second, the use of self-reported data to assess health professionals' practices may have predisposed study findings to social desirability bias, which may have caused reported practices to differ from actual behavior. A review of studies performed by Adams and others suggested that self-reporting of practices might overestimate actual behavior by up to 27%.^122^ Accordingly, there may have been an overestimation of the adherence to recommended practice guidelines. Third, the composite scores calculated for knowledge and practices were based on responses from just 10 questions. Fourth, some of the associations yielded may have been affected by self-selection biases. For example, although interactions between physicians and researchers were associated with higher knowledge scores, this result may have been affected by a selection bias, as physicians who choose to work with researchers may typically be more ambitious than those who do not. Finally, resource constraints, as well as limited participation in some countries (e.g., Mexico), in conducting this study prevented the collection of completely representative samples of health professionals in the four countries. As there was variation among sampling frames and a limited sample size, results from the study cannot, and should not, be compared across countries. It should also be noted that these variations may have also affected within-country results and that the same survey using a representative sample may have yielded different results.

### Policy implications.

Health professionals serve as a principal source through which people learn about and use health interventions, and have great influence over their patients' health-related behaviors.^1^,^123^–^127^ Recognizing the impact that clinicians can have on their patients, there is a pressing need to undertake efforts to improve health professionals' knowledge and practices. Such interventions may provide a practical and feasible opportunity to enhance treatment and control of diseases like tuberculosis worldwide. With the identification of gaps existing in knowledge and practices related to tuberculosis treatment, appropriate interventions addressing them through targeting health professionals can, and should, be implemented. In this regard, it is possible that relatively small investments focused on health professionals may result in considerable knowledge gains across large populations.^34^ There exist several studies on the means of bringing about behavioral change among health professionals that could be used to inform potential interventions. Examples include, but are not limited to, systematic reviews of studies on the effectiveness of audit and feedback,^128^ distribution of education materials,^129^ educational meetings,^130^ local opinion leaders,^131^ outreach visits,^132^ reminders,^133^ and the Health Systems Evidence database containing these types of reviews.^134^

### Future Research.

Future investigations building on the current study should aim to include representative samples of participants such that generalizability of the findings may be enhanced. Specifically, a larger sample of health professionals should be surveyed so that study populations can be nationally representative and participant groups can be compared with each other to allow for appropriate subgroup analyses. In addition, while the current study has identified several factors that may be associated with knowledge and practice gaps, more work is needed to identify, propose, and test new interventions that may address these gaps and evaluate those that already exist. Areas in which to explore intervention strategies include supporting health professionals' involvement in research activities and the training of health professionals. For example, one factor significantly associated with better practices of health professionals was training (since most recent degree completion) in the care of tuberculosis patients. In addition, further training around acquiring and critically appraising systematic reviews might enhance trust in systematic reviews of randomized controlled double-blind trials, which was a factor found to be associated with better practices among participants.

## Conclusions

There remains a clear need for increased efforts directed toward bridging the gap in tuberculosis treatment that exists between knowledge from research evidence and the current practices of health professionals. Multifaceted, targeted interventions are necessary to not only take advantage of evidence-informed therapies and practices, but also to halt those ineffective and ill-informed practices that only serve to perpetuate the burden of tuberculosis globally. Key agencies and authorities such as policymakers, civil society leaders, donors, and international organizations that aim to improve the treatment of tuberculosis should consider strategies that may be implemented to refine health professionals' knowledge and practices in this area. Targeted strategies may include facilitating and supporting interactions with researchers, offering training on acquiring and understanding systematic reviews, and providing further support for health professionals wishing to specialize in their practice. Further studies are required to confirm the present exploratory analyses and to further define directions for policy implementation.

## Figures and Tables

**Table 1:** Country profiles in 2005

	China	India	Iran	Mexico	Source
Population (in millions)	1,323	1,103	70	107	56
GDP per capita (in PPP international dollar)	6771	3,412	8,018	10,626	57
Per capita total expenditure on health (in PPP international dollar)	277	91	604	655	58
Per capita government expenditure on health (in PPP international dollar)	105	16	288	304	59
Life expectancy at birth for males/females (in years)	71/74	62/65	68/71	72/77	59
Children under-five mortality rate (per 1,000 live births)	27	56	36	27	59
Prevalence of active tuberculosis (per 100,000 population)	208	299	30	27	59
Personal computers per population (%, 2004)	4	3	11	11	59
Internet users per population (%, 2004)	7	1	9	13	59

Data are for 2005 unless otherwise indicated; PPP = purchasing power parity.

**Table 2:** Descriptive statistics on health professionals' individual characteristics, working context, and views about and use of research evidence

Factor	All (*N* = 384)	China (*N* = 116)	India (*N* = 100)	Iran (*N* = 108)	Mexico (*N* = 60)
Individual characteristics					
Age, mean (years)	39.8	37.2	43.2	34.7	48.1
Sex, male	65.0	82.8	37.4	71.3	65.0
Type of health professional					
General practitioner	80.8	72.4	91.8	87.0	68.3
Specialist physician	9.7	7.8	1.0	13.0	21.7
Nurse	1.3	0.9	4.1	0	0
Health worker	6.3	19.0	2.1	0	0
Other	1.8	0	1.0	0	10.0
Allocation of time (% of time)†					
Clinical practice	63.3	65.5	72.7	51.0	65.0
Research	4.6	6.3	0	3.2	11.3
Teaching	10.1	3.9	7.2	18.5	12.2
Administration	16.5	12.2	20.6	22.2	7.5
Master's or doctorate degree	4.2	1.7	1.0	2.8	16.7
Training since completed last degree					
Acquiring systematic reviews through the Cochrane Library	3.0	0	0	2.9	15.7
Critically appraising systematic reviews	5.9	7.0	1.1	2.9	17.6
Care of patients with tuberculosis	78.9	91.4	96.7	61.9	55.4
Easy access to personal computer with CD ROM (vs. less easy, not easy, no access, or not sure)	21.7	21.7	1.1	26.9	50.0
Easy access to Internet (vs. less easy, not easy, no access, or not sure)	19.7	18.3	1.1	24.1	48.9
Able to read and write English well or very well (vs. little or no ability)	52.3	16.4	98.0	50.9	48.3
Practice*					
Operating authority of facility or practice					
Government	95.0	93.1	96.0	97.2	93.3
Nongovernmental organization	5.5	7.8	4.0	3.7	6.7
For-profit organization	2.6	0	0	9.3	0
Type of facility or practice					
Solo or individual practice	15.2	3.4	23.2	13.0	28.8
Group practice	4.2	1.7	0	5.6	13.6
Hospital	24.1	38.8	14.1	18.5	22.0
Community health center	55.2	9.5	80.8	81.5	54.2
Location of facility or practice					
Urban	49.6	10.3	96.0	29.9	83.3
Rural	36.3	81.0	2.0	39.3	1.7
Mixed	14.1	8.6	2.0	30.8	15.0
Facility had anti-tuberculosis drugs available	75.9	29.3	100.0	95.4	91.5
Views and activities related to improving clinical practice					
Research performed in their own country is of above average or excellent quality	55.7	77.6	37.0	37.4	75.0
Trust somewhat or completely a systematic review of randomized controlled double-blind trials	52.5	37.9	54.1	54.2	75.9
Working with researchers or research groups to improve clinical practice or the quality of working life	24.1	42.2	6.3	16.8	31.0
Higher quality of available research is important or very important to improve their work	77.5	76.7	59.6	85.2	94.9
Used or read particular sources of evidence					
Clinical practice guidelines, protocols or decision-support tools	54.7	76.5	48.0	26.2	74.5
Cochrane Library	5.5	0.9	0	5.6	26.8
Scientific journals from high-income countries	17.9	0.9	4.5	23.1	64.0
Scientific journals from own country	58.3	67.8	41.6	47.6	81.8
Summaries of articles, reports, and reviews from public and not-for-profit health organizations	55.8	46.1	54.4	59.8	71.2

Note that because of variations among sampling frames and a limited sample size, these results cannot, and should not, be compared across countries.

* May not add to 100% because health professional may practice in more than one setting.

† May not add to 100% because the allocation of time reported by a small number of respondents did not add to 100%.

**Table 3:** Questions assessing health professionals' knowledge about treating tuberculosis

Question (multiple choice)	Answer (A, B, C, D, E, F)	All (*N* = 384)	China (*N* = 116)	India (*N* = 100)	Iran (*N* = 108)	Mexico (*N* = 60)
1) What factors accelerate the progression from tuberculosis infection to disease?	(A) Malnutrition [T]	95.3%	91.4%	96.0%	98.1%	96.7%
	(B) HIV infection [T]	95.6%	91.4%	93.0%	100.0%	100.0%
	(C) Diabetes [T]	63.5%	63.8%	35.0%	76.9%	86.7%
	(D) Long-term treatment with corticosteroids or immunosuppressive medications [T]	80.7%	96.7%	34.0%	99.1%	95.0%
	(E) Bacillus Calmette-Guerin (BCG) vaccination [F]	26.6%	69.8%	3.0%	6.5%	18.3%
	(★) Correctly answered A, B, C, D, and not E	43.5% (167/384)	19.8% (23/116)	27.0% (27/100)	71.3% (77/108)	66.7% (40/60)
2) All of the following are first line therapy for TB except:	(A) Isoniazid [F]	9.4%	0%	0%	15.7%	31.7%
	(B) Rifampin [F]	8.9%	0%	0%	15.7%	28.3%
	(C) Streptomycin [F]	10.4%	1.7%	2.0%	16.7%	30.0%
	(D) Cycloserine [T]	89.3%	99.1%	99.0%	82.4%	66.7%
	(E) Ethambutol [F]	9.6%	0%	0%	16.7%	31.7%
	(★) Correctly answered D and not A, B, C, E	87.2% (335/384)	98.3% (114/116)	98.0% (98/100)	78.7% (85/108)	63.3% (38/60)
3) The major side effect of isoniazid therapy is:*	(A) Gastritis [F]	21.3%	12.9%	49.0%	1.9%	27.8%
	(B) Hepatitis [T]	66.5%	87.9%	25.5%	88.9%	50.0%
	(C) Diarrhea [F]	4.5%	7.8%	4.1%	0.9%	5.6%
	(D) Tubular necrosis [F]	12.0%	6.9%	31.6%	0.9%	9.3%
	(E) Optic neuritis [F]	23.4%	52.6%	7.1%	9.3%	18.5%
	(★) Correctly answered B and not A, C, D, E	45.4% (171/376)	34.5% (40/116)	13.3% (13/98)	87.0% (94/108)	44.4% (24/54)
4) Which one of the following is the best indicator of a patient not being infectious?†	(A) Patient has received at least 2 weeks of TB medications [F]	12.8%	2.6%	2.0%	37.7%	6.7%
	(B) Patient has no cough [F]	4.7%	4.3%	1.0%	7.5%	6.7%
	(C) Patient has three negative acid-fast bacilli (AFB) sputum smears on three consecutive days [T]	83.8%	94.8%	97.0%	55.7%	90.0%
	(★) Correctly answered C and not A, B	82.7% (316/382)	93.1% (108/116)	97.0% (97/100)	54.7% (58/106)	88.3% (53/60)
5) The minimum duration of therapy for culture-proven active TB is:	(A) 2 weeks [F]	0.3%	0.9%	0%	0%	0%
	(B) 6 weeks [F]	3.7%	6.9%	1.0%	2.8%	3.4%
	(C) 2 months [F]	2.9%	0%	1.0%	3.7%	10.2%
	(D) 6 months [T]	87.7%	94.8%	80.6%	92.6%	76.3%
	(E) 9 months [F]	4.5%	0%	16.3%	0%	1.7%
	(F) 12 months [F]	2.1%	0%	1.0%	0.9%	10.2%
	(★) Correctly answered D and not A, B, C, E, F	86.9% (331/381)	92.2% (107/116)	80.6% (79/98)	92.6% (100/108)	76.3%(45/59)
All five questions	Answered correctly	11.7% (45/384)	5.2% (6/116)	5.0% (5/100)	19.4% (21/108)	21.7% (13/60)

[T] = true, [F] = false. Data show the percentage and fraction of respondents who correctly answered each question. Note that because of variations among sampling frames and a limited sample size, these results cannot, and should not, be compared across countries.

* In Chinese, the words “is” and “are” are the same. Chinese health professionals, therefore, were disadvantaged in answering this question as they were not told that there is only one major side effect of isoniazid therapy (as indicated by the word “is” in English as compared with “are”). This language difference likely explains why many Chinese health professionals circled more than one answer for this question, while health professionals from other countries consistently circled only one answer. The proportion reported in this study, therefore, shows the number of Chinese professionals who circled the correct answer, regardless of whether incorrect answers were also circled.

† Practice guidelines in Iran are different from the WHO guidelines that were used as correct answers for this test question.

**Table 4:** Questions assessing health professionals' practices relating to treating tuberculosis

Question (multiple choice)	Answer (frequency)	All (*N* = 384)	China (*N* = 116)	India (*N* = 100)	Iran (*N* = 108)	Mexico (*N* = 60)
1) When treating new active tuberculosis patients, how often did the provider prescribe a treatment regimen of 5 months or less? [Contrary to recommended practice]	(A) Never	72.6%	89.7%	65.7%	81.5%	35.0%
	(B) Rarely	9.7%	0.9%	24.2%	3.7%	13.3%
	(C) Sometimes	3.9%	0%	8.1%	2.8%	6.7%
	(D) Often	1.8%	0%	1.0%	0%	10.0%
	(E) Very often	2.9%	0%	1.0%	1.9%	15.0%
	(F) N/A	9.1%	9.5%	0%	10.2%	20.0%
	Never or rarely	82.3%	90.6%	89.9%	85.2%	48.3%
2) When treating new active tuberculosis patients, how often did the provider (or someone acting on their behalf) ensure that the treatment was taken for at least 2 months in the presence of a health worker? [Recommended practice]	(A) Never	5.5%	9.6%	0%	3.7%	10.0%
	(B) Rarely	3.4%	0%	0%	9.3%	5.0%
	(C) Sometimes	3.9%	2.6%	1.0%	8.3%	3.3%
	(D) Often	24.5%	29.6%	11.0%	35.2%	18.3%
	(E) Very often	56.4%	49.6%	88.0%	39.8%	46.7%
	(F) N/A	6.3%	8.7%	0%	3.7%	16.7%
	Often or very often	80.9%	79.2%	99%	75%	65%
3) Before initiating treatment with new active tuberculosis patients, how often did the provider (or someone acting on their behalf) provide health education on the importance of taking medication regularly? [Recommended practice]	(A) Never	2.4%	0.9%	1.0%	1.9%	8.3%
	(B) Rarely	1.0%	0%	0%	3.7%	0%
	(C) Sometimes	3.4%	2.6%	0%	4.6%	8.3%
	(D) Often	24.0%	40.9%	10.0%	22.2%	18.3%
	(E) Very often	64.2%	49.6%	89.0%	64.8%	50.0%
	(F) N/A	5.0%	6.1%	0%	2.8%	15.0%
	Often or very often	88.2%	90.5%	99%	87%	68.3%
4) When treating new active tuberculosis patients, how often did the provider (or someone acting on their behalf) notify the health authority about the new patients? [Recommended practice]	(A) Never	4.2%	3.5%	0%	6.5%	8.3%
	(B) Rarely	2.1%	2.6%	0%	2.8%	3.3%
	(C) Sometimes	2.9%	4.4%	0%	4.6%	1.7%
	(D) Often	18.0%	35.7%	12.0%	6.5%	15.0%
	(E) Very often	67.4%	49.6%	87.0%	74.1%	56.7%
	(F) N/A	5.5%	4.4%	1.0%	5.6%	15.0%
	Often or very often	85.4%	85.3%	99%	80.6%	71.7%
5) When treating HIV-infected individuals, how often did the provider recommend preventive chemotherapy for tuberculosis? [Recommended practice]	(A) Never	31.1%	9.5%	45.0%	44.9%	25.0%
	(B) Rarely	2.6%	0%	4.0%	1.9%	6.7%
	(C) Sometimes	2.9%	1.7%	4.0%	1.9%	5.0%
	(D) Often	6.5%	1.7%	13.0%	2.8%	11.7%
	(E) Very often	12.0%	0%	27.0%	5.6%	21.7%
	(F) N/A	44.9%	87.1%	7.0%	43.0%	30.0%
	Often or very often	18.5%	1.7%	40%	8.4%	33.4%
All recommended practices	Followed often or very often	3.1% (12/384)	0% (0/116)	7.0% (7/100)	1.9% (2/108)	5.0% (3/60)

Data show the percentage and fraction of respondents who over the previous 12 months engaged in the recommended practices described in the first four questions either often or very often (vs. never, rarely, sometimes, and not applicable) and who never engaged in the non-recommended practice as described in the last question (vs. rarely, sometimes, often, very often, and not applicable). Note that because of variations among sampling frames and a limited sample size, these results cannot, and should not, be compared across countries.

**Table 5:** Ordinal logistic models for the factors associated with the log odds of having higher knowledge and better practices

Factor	Knowledge (*N* = 340)		Practices (*N* = 340)	
	OR	95% CI	OR	95% CI
Individual and practice characteristics				
Age*	1.02	(0.82, 1.28)	0.97	(0.78, 1.21)
Age squared*	1.00	(1.00, 1.00)	1.00	(1.00, 1.00)
Sex, male	0.75	(0.37, 1.56)	0.84	(0.53, 1.31)
Specialist physician	**2.84**	**(1.15, 7.05)**	2.19	(0.95, 5.07)
Time allocated to research†	1.00	(1.00, 1.01)	1.00	(0.97, 1.03)
Master's or doctorate degree	1.16	(0.48, 2.78)	0.74	(0.23, 2.38)
Training (since completed last degree) in:				
Acquiring systematic reviews through the Cochrane Library	0.82	(0.44, 1.54)	1.94	(0.88, 4.26)
Critically appraising systematic reviews	0.70	(0.36, 1.35)	1.37	(0.51, 3.68)
The care of patients with tuberculosis	1.15	(0.73, 1.80)	**2.63**	**(1.05, 6.60)**
Easy access to the internet	1.11	(0.76, 1.63)	0.75	(0.22, 2.62)
Able to read and write English well or very well	0.86	(0.31, 2.39)	1.02	(0.71, 1.47)
Working context				
Based in a facility or practice with an NGO as the operating authority	0.65	(0.19, 2.25)	0.78	(0.36, 1.71)
Located in an urban setting	0.93	(0.45, 1.90)	1.36	(0.91, 2.01)
Based in a hospital	0.73	(0.42, 1.26)	**1.35**	**(1.18, 1.54)**
Facility had anti-tuberculosis drugs available	0.67	(0.41, 1.10)	1.10	(0.26, 4.70)
Views and activities related to improving clinical practice				
Research performed in their own country is of above average or excellent quality	0.67	(0.26, 1.70)	0.98	(0.76, 1.25)
Trust somewhat or completely a systematic review of randomized controlled double-blind trials	0.95	(0.58, 1.56)	**1.78**	**(1.36, 2.35)**
Working with researchers or research groups to improve clinical practice or the quality of working life	**1.48**	**(1.01, 2.17)**	1.08	(0.82, 1.42)
Higher quality of available research is important or very important to improve their work	0.62	(0.37, 1.04)	1.26	(0.89, 1.78)
Used or read particular sources of evidence				
Clinical practice guidelines, protocols or decision-support tools	0.93	(0.50, 1.72)	1.37	(0.59, 3.16)
Cochrane Library	0.81	(0.16, 4.11)	0.79	(0.24, 2.54)
Scientific journals from high-income countries	0.58	(0.21, 1.63)	0.60	(0.18, 2.01)
Scientific journals from own country	1.14	(0.65, 1.98)	1.46	(0.80, 2.64)
Summaries of articles, reports, and reviews from public and not-for-profit health organizations	0.82	(0.59, 1.15)	1.42	**(1.05, 1.92)**
Thresholds				
k1	−1.59	(−5.81, 2.62)	−0.27	(−4.32, 3.77)
k2	−0.36	(−4.66, 3.95)	1.11	(−2.95, 5.17)
k3	0.28	(−3.95, 4.50)	2.24	(−1.89, 6.38)
k4	1.72	(−2.51, 5.95)	3.55	(0.00, 7.09)

CI = confidence interval; NGO = nongovernmental organization; OR = odds ratio. Standard errors adjusted for four clusters (i.e., countries). All regression models include country dummies (China is the reference country). Bolded entries were statistically significant factors in the models.

* Entered in regression models as continuous variables measured in years.

† Entered in regression models as continuous variable measured in percent of time (0–100).
